# Persistent Hypokalemia in a Patient With Ogilvie’s Syndrome

**DOI:** 10.7759/cureus.32056

**Published:** 2022-11-30

**Authors:** Tsering Dolkar, Samaj Adhikari, Pooja Devi, Sandra O Nwani, Muhammad Dogar

**Affiliations:** 1 Internal Medicine, One Brooklyn Health System Interfaith Medical Center, Brooklyn, USA; 2 Internal Medicine, One Brooklyn Health System Interfaith Medical Center, New York, USA; 3 Cardiology, One Brooklyn Health System Interfaith Medical Center, Brooklyn, USA

**Keywords:** neostigmine, refractory hypokalemia, hypokalemia, ogilvie's syndrome, acute colonic pseudo-obstruction

## Abstract

Ogilvie's syndrome, also known as acute colonic pseudo-obstruction (ACPO), is a rare disease characterized by acute dilatation of the colon in the absence of anatomic intestinal obstruction. It is of clinical importance because of its preponderance in elderly males in the seventh decade of life who may present with constipation or diarrhea. We present an 80-year-old male who presented with diarrhea, with laboratory investigations showing hypokalemia and a CT abdomen revealing colonic distension. The patient was wasting potassium both from colon and renal losses, despite low aldosterone levels. The patient was treated with Neostigmine, which helped relieve abdominal distention. Subsequently, potassium was corrected with aggressive replacement. This case sheds light on newer modalities of treatment such as neostigmine, as in this case.

## Introduction

Ogilvie syndrome (OS) is cited as one of the rare diseases in the literature, with approximately 100 cases per 100,000 hospital admissions annually [1]. However, making the diagnosis and the basis for the diagnosis is sparingly done. It is, however, common in the elderly male population from the seventh decade and above. Also known as acute colonic pseudo-obstruction (ACPO), it is the acute dilation of the colon without any associated mechanical dilation. The most common phenotype in Ogilvie's syndrome is presumed to be associated with increased potassium wasting from the colon due to an increased expression of BK channels in the colonic mucosa with increased sensitivity to normal serum aldosterone levels [2]. In this case report, we discuss Ogilvie syndrome as a rare disease with recalcitrant and persistent hypokalemia from both colonic and renal wasting in a patient with low aldosterone levels.

## Case presentation

An 80-year-old male with a past medical history of hypertension, heart failure with reduced ejection fraction (HFrEF), diabetes mellitus, gastroesophageal reflux disease (GERD), and dementia was brought from a nursing home to the emergency room (ER) due to low potassium (2.0 mg/dl) and abdominal distension. The patient also had an asymptomatic COVID-19 infection during this admission. The patient had a history of abdominal distention with recurrent hypokalemia during the previous admissions.

An extensive workup for the causes of abdominal distention was done to rule out obstruction via imaging and laboratory investigations, including the workup for hyperaldosteronism, Cushing’s disease, and Clostridium difficile infection, all of which were negative (Table 1). Further laboratory investigations showed hypokalemia with kalikuria. Pertinent labs are shown in Table 1.

**Table 1:** Relevant laboratory findings. WBC: white blood cells, BUN: blood urea nitrogen, ACTH: adrenocorticotropic hormone, TSH: thyroid-stimulating hormone, T4: thyroxine.

Investigations	Values	Reference
Hemoglobin	9.9	13.0–17.0 g/dL
Hematocrit	31.6	39–53%
WBC	4.5	4.5–11.0 × 10 × 3/µL
Platelets	302	130–400 × 10 × 3/µL
Glucose	94	80–115 mg/dL
BUN	13.2	8.4–25.7 mg/dL
Creatinine	0.73	0.72–1.25 mg/dL
Sodium	144	136–145 mmol/L
Potassium	2.0	3.5–5.1 mmol/L
Chloride	107	98–107 mmol/L
Bicarbonate	26	23–31 mmol/L
Calcium	8.6	8.8–10.0 mg/dL
Albumin	3.3	3.2–4.6 g/dL
Magnesium	1.9	1.6–2.6 mg/dL
Renin	<0.167	0.167–5.380 ng/ml/hr
Aldosterone	<1.0	0.0-30.0 ng/dl
ACTH	14.5	7.2-63.3 pg/ml
Aldosterone/renin	Unable to calculate	ng/dl per ng/ml/hr
TSH	0.367	0.465–4.680 uIU/ml
Free T4	1.67	0.78–2.19 ng/dl
Urine sodium	93.3	3.5-5.1 mmol/l
Urine potassium	16.4	3.5–5.1 mmol/l
Stool potassium	150	<100 mmol/L
*Clostridium difficile* toxin gene	Negative	N/A
WBC stool	None detected	N/A

During the previous admission, the patient was started on losartan for hypertension, and spironolactone was added, which corrected and maintained the potassium levels, but the patient had hyperkalemia of 6.4 mmol/l. As such, spironolactone was discontinued.

**Figure 1:**
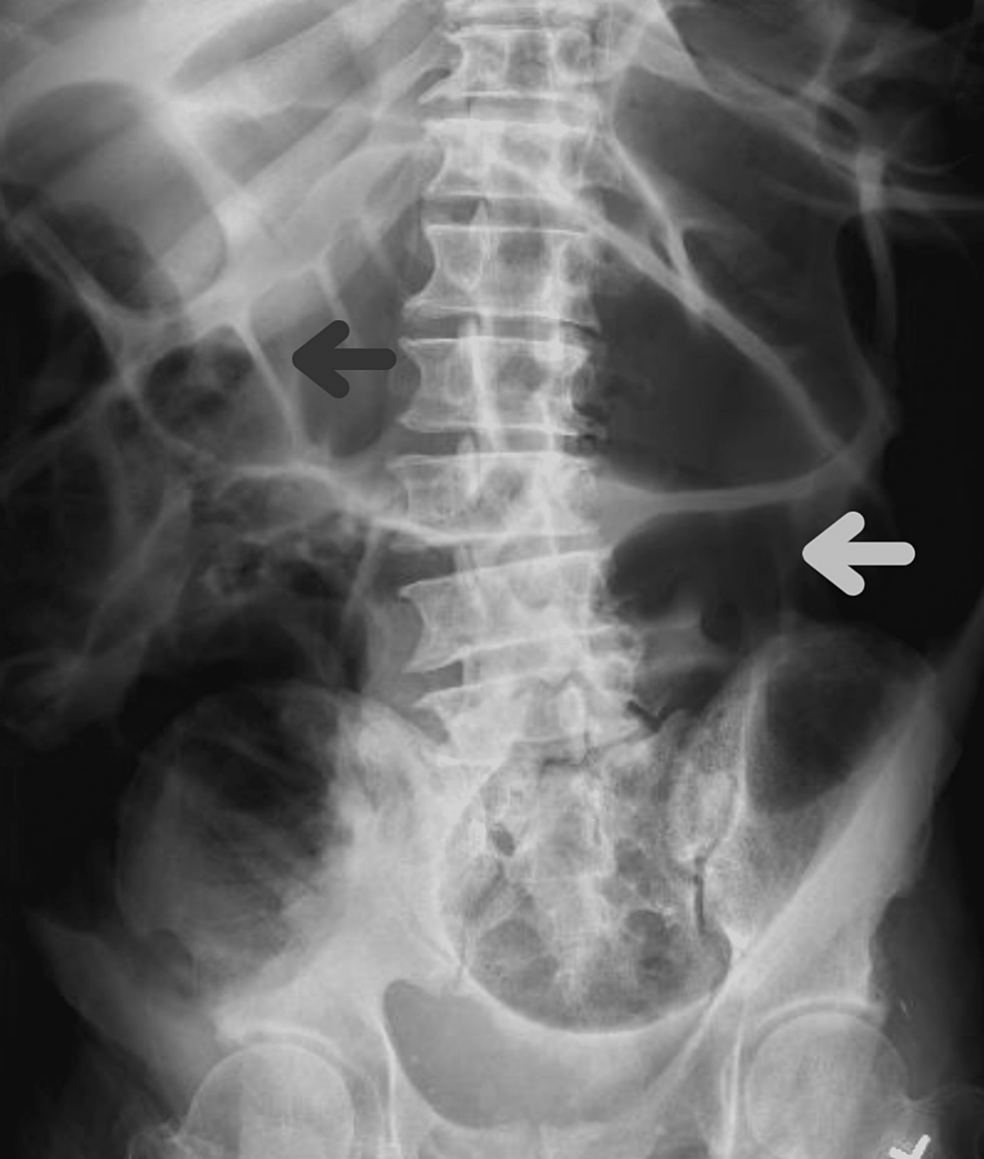
X-ray of the abdomen in flat and upright position. There are numerous distended bowel loops throughout the visualized abdomen. Limited evaluation for intraperitoneal free air on the provided view with no abnormal calcifications.

**Figure 2:**
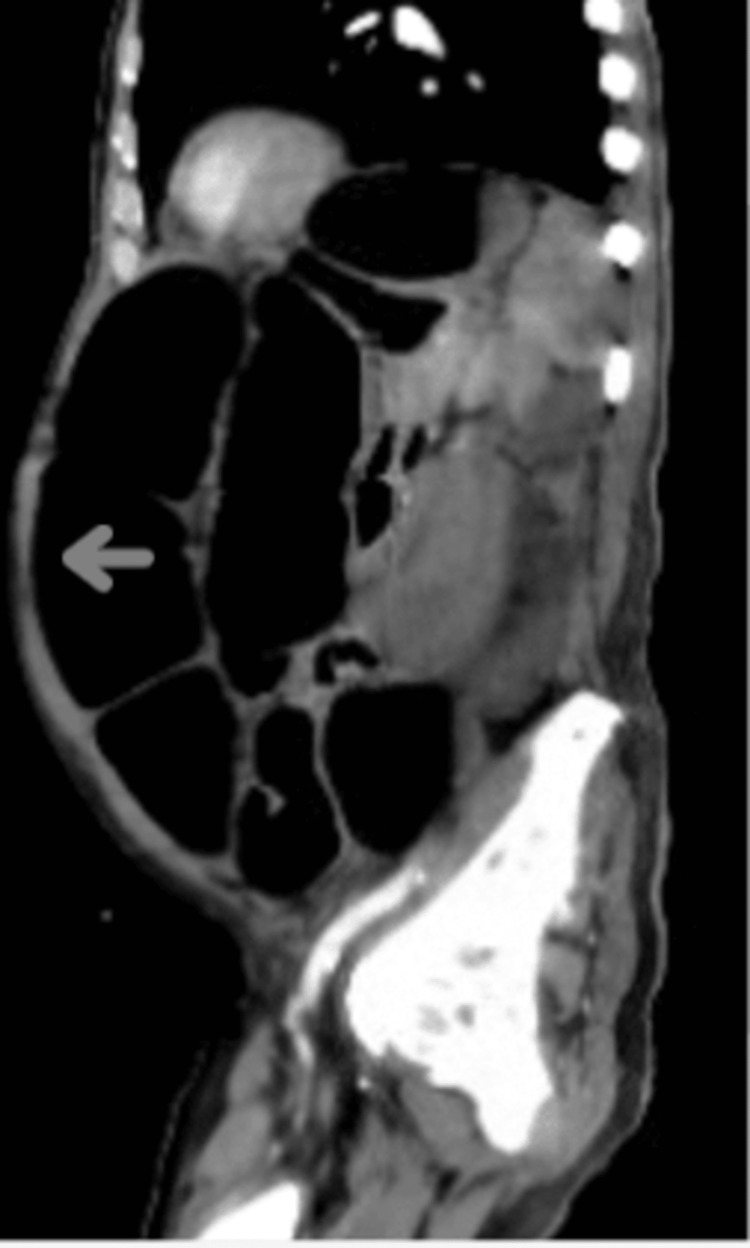
CT scan sagittal view showing marked gaseous distention of the colon representing megacolon. Marked gaseous distention of the colon, which may represent a megacolon. No obvious obstructing lesion is identified.

**Figure 3:**
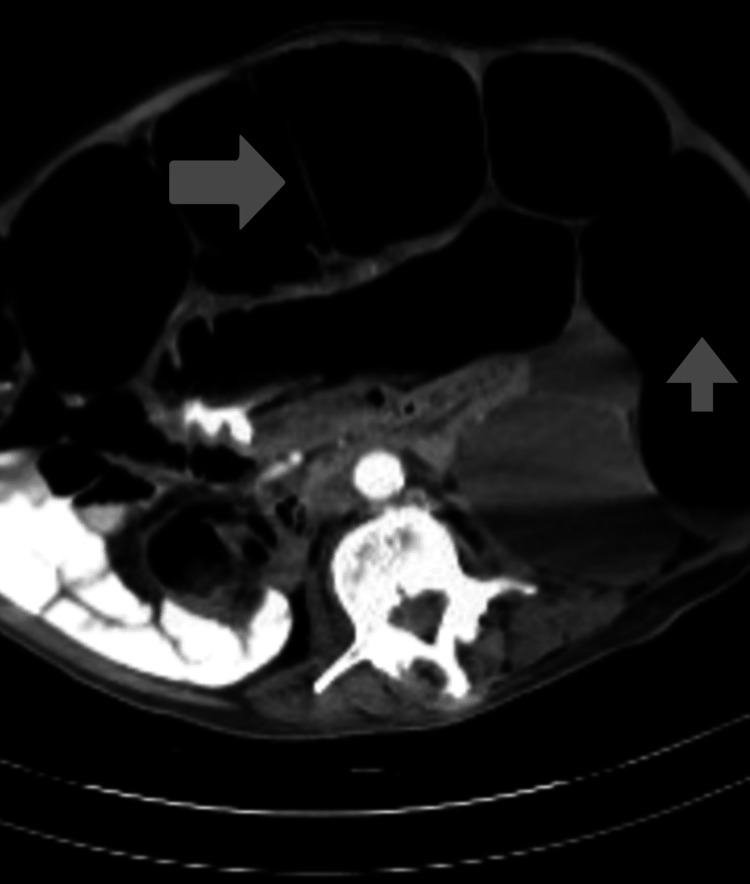
CT scan transverse view of the abdomen. CT scan transverse view showing marked gaseous distention of the colon, which may represent a megacolon. No obvious obstructing lesion noted.

A plain abdominal X-ray (Figure 1) revealed numerous distended bowel loops throughout the abdomen with differential diagnoses of obstruction and ileus. The CT abdominal sagittal view (Figure 2) and transverse view (Figure 3) showed marked gaseous distention of the colon, which may represent a megacolon with no obvious obstructing lesion.

Due to the long-standing abdominal distention and failed conservative measures, a fleet enema and lactulose were given. A nasogastric (NG) and rectal tube were inserted with immediate relief of the abdominal distention, and electrolytes were supplemented. Medication histories were reviewed extensively to rule out the history of previous or current use of neuroleptic medications, anticholinergics, steroids, amphetamines, and narcotics to rule out drug-induced hypokalemia.

Neostigmine 2 mg IV over 3-5 min was given on day 8 of the admission, and the abdominal distention resolved. However, the patient still had refractory hypokalemia. Potassium was replaced, and the patient was restarted on spironolactone.

It is important to note that a colonoscopy for the abdominal distention was considered but held due to this patient's severe and refractory hypokalemia. The abdominal distention subsided with the presence of bowel sounds. Subsequently, a carbohydrate-controlled diet was started.

The patient was upgraded to continuous cardiac monitoring due to hypokalemia, even with correction and bradycardia in the 40s. The bradycardia was initially managed with a transcutaneous pacemaker and subsequently with a transvenous pacemaker.

## Discussion

Colonic pseudo-obstruction is characterized by massive colon dilatation in the absence of mechanical obstruction or toxic megacolon, profuse watery diarrhea, and stools with low sodium and high potassium levels, which can be seen in several medical and surgical conditions. It is thought to be caused by a distal colonic imbalance of sympathetic and parasympathetic input. ACPO should be differentiated from colonic obstruction and toxic megacolon, which require blood tests and radiological tests, e.g., a plain abdominal radiograph, abdominal computed tomography (CT), and a water-soluble contrast enema to detect the complications.

The phenotype associated with secretory diarrhea is rare. It is related to increased potassium channel activity in the colon, inducing excessive potassium loss with increased sensitivity to normal serum aldosterone levels. Colonic pseudo-obstruction results in extrarenal potassium loss [3]. Patients who are affected frequently lose more than 100 mmol of potassium daily. High potassium losses in the stool cause persistent hypokalemia and take a longer time to improve despite aggressive repletion. This is most likely mediated by increased BK channel expression in the colonic mucosa. Aldosterone is thought to play a role in regulating BK channels [4].

Patients with acute colonic pseudo-obstruction are initially managed by supportive care, which includes treatment of the underlying causes like infection and congestive heart failure, discontinuation of offending medications, optimization of electrolytes and pressure with intravenous fluids, and decompression of the gastrointestinal tract with the placement of a nasogastric tube [5]. In most uncomplicated cases, there is resolution of colonic dilatation.

Clinical and radiological controls at close intervals are required until the condition is resolved. If patients do not respond within one to two days or if ACPO has already reached a critical duration (>3-4 days) or extent (i.e., cecal diameter ≥12 cm), Neostigmine should be administered, which leads to long-lasting success in approximately three out of four patients [4]. Patients who are still refractory to treatment should receive endoscopic decompression. More invasive therapeutic options, such as a cecostomy or (segmental) colonic resection, should only be considered for patients who do not respond to conservative treatment [4].

The main prognostic factors are age, associated diseases, elapsed time and diameter of cecal dilatation, and necrosis and perforation. The recurrence rate after medical treatment is 20-50%, and intrahospital mortality is 30% [6]. Our case was admitted to the emergency department with abdominal distension and diarrhea and laboratory findings significant for severe hypokalemia (stool K in our case, 150 mmol/L), which corroborates the result of acute pseudocolonic obstruction. The urine potassium to creatinine ratio (K/Cr) is typically <13 meq/g. Our patient had urine K/Cr of 16.98 mEq/g, which is slightly higher than expected, given the significant potassium (K) loss from stool should have conserved K from the kidneys.

According to Valle et al., 127 patients with acute colonic pseudo-obstruction were treated with Neostigmine in four randomized trials, of which 65 were given the drug. Only one dose of Neostigmine effectively resolved acute colonic pseudo-obstruction compared with a placebo [7]. Doses of 2-5 mg of neostigmine should be administered slowly by intravenous injection over five minutes. Continuous vital signs and electrocardiogram monitoring should be performed 30 minutes after injection [8]. Neostigmine is recommended in patients suffering from acute pseudo-obstructions of the colon or those who have failed conservative treatment after 48 to 72 hours. Neostigmine is relatively contraindicated in persons who have recently had a myocardial infarction, acidosis, asthma, bradycardia, or peptic ulcer disease and are taking beta-blockers [9].

Management with the administration of spironolactone has been previously reported in patients with relative hyperaldosteronism due to increased sensitivity of colonocytes to aldosterone [2]. Our patient received two doses of neostigmine, which helped relieve the abdominal distension. Subsequently, serum potassium was corrected with aggressive replacement. There were no further losses, and the patient remained eukalemic.

## Conclusions

Acute colonic pseudoobstruction is a rare disease that needs prompt medical attention because it causes life-threatening persistent hypokalemia, which can be hard to treat with ongoing GI losses of potassium. Neostigmine is beneficial in refractory cases that fail symptomatic management. We recommend further studies for newer treatment modalities.
